# Exploring Gene–Diet Interactions for Mother–Child Health: A Systematic Review of Epidemiological Studies

**DOI:** 10.3390/nu16070994

**Published:** 2024-03-28

**Authors:** Giuliana Favara, Andrea Maugeri, Roberta Magnano San Lio, Martina Barchitta, Antonella Agodi

**Affiliations:** Department of Medical and Surgical Sciences and Advanced Technologies “GF Ingrassia”, University of Catania, 95123 Catania, Italy; giuliana.favara@unict.it (G.F.); andrea.maugeri@unict.it (A.M.); robertamagnanosanlio@unict.it (R.M.S.L.); martina.barchitta@unict.it (M.B.)

**Keywords:** mother–child health, pregnancy, interaction, genetic variants, diet, nutrition

## Abstract

Background: Maternal–child health suggests the critical impact of maternal nutrition during the pre-conception and gestational periods, with some genetic variants also playing a significant role. Our systematic review provides an overview of epidemiological studies exploring the interactions between genetic variants, maternal dietary habits, and neonatal and/or maternal pregnancy outcomes. Methods: From its inception until June 2023, we conducted a comprehensive literature search on PubMed, Embase, and Web of Science databases. Results: On a total of 29 epidemiological studies, 11 studies were conducted to explore the interplay between genetic variants and dietary factors, focusing on the risks associated with gestational diabetes mellitus, hypertensive disorders of pregnancy, recurrent spontaneous abortion, recurrent pregnancy loss, iron deficiency anemia, and gestational weight gain. Concerning neonatal outcomes, six studies investigated the interplay between genetic variants, dietary factors, and anthropometric measures, while eight studies delved into abnormal embryonic development, two studies focused on preterm birth, and two studies explored other neonatal outcomes. Conclusions: Deeply understanding gene–diet interactions could be useful in developing highly personalized approaches to maternal and child nutrition, as well as in exploring the potential implications in disease prevention and the promotion of the long-term well-being of both mothers and their offspring.

## 1. Introduction

Maternal–child health is a significant focus in the worldwide realm of public health, with maternal–child health holding paramount importance. The impact of the health of both mothers and infants during the pre-conceptional and gestational periods is profound, exerting enduring effects on the health of future generations. Maternal nutrition emerges as one of the most critical factors during these periods, influencing the well-being of women and playing a pivotal role in the proper development and growth of newborns [1,2].

Certainly, there is evidence suggesting that the dietary habits of expectant mothers might impact the probability of encountering adverse pregnancy outcomes, including gestational diabetes mellitus (GDM), pre-eclampsia, intrauterine growth restriction, low birth weight (LBW), and being born small for gestational age (SGA) [3,4,5]. Other factors can play a role in promoting a healthy pregnancy, such as achieving appropriate weight gain, engaging in physical activity, and incorporating mineral and vitamin supplements [6,7]. In general, ensuring a sufficient intake of specific nutrients (such as folate and vitamin D) before conception and throughout pregnancy is essential for the correct development of both the placenta and the fetus, contributing to a sustained path of long-term health [8,9,10,11,12].

Pregnancy involves an intricate interplay that originates from the interaction of maternal diet and genetics, coupled with fetal genetic information and the availability of nutrients in utero [13,14,15,16]. The significance of genetic susceptibility is widely acknowledged, encompassing recently identified genetic variants in both the fetus and the mother. These variants, in consequence, influence the intrauterine environment, the duration of pregnancy, and fetal growth. For example, a multitude of studies have presented evidence highlighting the noteworthy associations between genetic variations in key enzyme genes related to the folate metabolic pathway (e.g., methylenetetrahydrofolate reductase, MTHFR) and an elevated risk of adverse outcomes [17].

Other studies have suggested a possible association between vitamin D receptor (VDR) polymorphisms and the likelihood of experiencing adverse pregnancy outcomes, including preterm birth (PTB), LBW, and SGA births [18,19,20]. For these reasons, maternal genetic diversity can exert profound effects on an individual’s response to dietary factors, altering the metabolism of nutrients, modulating susceptibility to specific health conditions, and ultimately influencing neonatal outcomes. Hence, exploring the complexities inherent in the interplay between genetics and diet holds the potential to revolutionize our approach to maternal–child health. This endeavor facilitates the design of precise, individualized strategies for maternal and child nutrition. While the intricate connection among genetics, diet, and maternal–child health forms a multifaceted tapestry deserving exploration, only a restricted number of studies have delved into the repercussions of the interactions between folate-related genes and nutrients on fetal growth outcomes. This limited research has produced results that, at times, diverge and generate controversy [21,22,23].

Within this framework, the present systematic review of epidemiological studies aims to delve into the intricate interplay among maternal nutrition, genetic variations, and outcomes pertaining to both mothers and neonates. By examining a wide spectrum of epidemiological evidence, the review aspires to comprehensively understand how these factors dynamically interact and influence the overall health trajectories during and after pregnancy.

## 2. Materials and Methods

### 2.1. Literature Search

From its inception until June 2023, we conducted a comprehensive literature search across the PubMed, Embase, and Web of Science databases. The primary focus of this search was to identify epidemiological studies that explored the interactions between genetic variants, maternal dietary habits, and neonatal and/or maternal pregnancy outcomes.

Two authors (G.F and A.M) independently carried out the literature search using the following terms: (Diet* OR Food* OR Nutrient* OR Vitamin* OR Mineral* OR Macronutrient* OR Micronutrient*) AND (Mutation* OR Polymorphism* OR SNP OR SNPs OR “Genetic variation” OR “Gene variant*”) AND (Gravidity OR Pregnan* OR Neonat* OR Infant* OR Mother* OR Maternal) AND (Interact* OR Modif*). The methodology of the current systematic review followed the guidelines outlined in the Preferred Reporting Items for Systematic Reviews and Meta-analyses (PRISMA) statement, as well as the recommendations provided in the Cochrane Handbook [24]. The PRISMA checklist is reported as a Supplementary Materials.

### 2.2. Selection Criteria

The authors independently chose the studies they retrieved based on the following selection criteria: (i) articles published in the English language, describing (ii) observational epidemiological studies, (iii) conducted on pregnant women and/or their offspring, (iv) that assessed maternal and/or neonatal genetic variations, (v) and examined their interaction with maternal dietary habits and/or nutrient intake (vi) in relation to maternal and/or neonatal outcomes.

Conversely, the following types of documents were excluded: (i) abstracts lacking full-text content and articles not in English; (ii) case reports or case series; (iii) comments, letters, editorials, and reviews; (iv) unpublished studies; (v) studies involving pediatric patients or non-pregnant women; (vi) studies conducted using in vitro or animal research models; (vii) and those not investigating the interplay between genetic variations, maternal exposures, and pregnancy outcomes.

### 2.3. Data Extraction

From each eligible study, the authors independently extracted the following information using a structured format: first author’s last name, year of publication, study design, country, study population, dietary factors, genetic variants, molecular approach used, biological sample, and outcome considered. With respect to maternal pregnancy outcomes, we considered GDM, hypertensive disorders of pregnancy (HDP), recurrent spontaneous abortion (RSA), recurrent pregnancy loss (RPL), iron deficiency anemia (IDA), and gestational weight gain (GWG). Neonatal outcomes included anthropometric measures, preterm birth, and abnormal embryonic development. During the study selection and data extraction phases, any inconsistencies between G.F and A.M were resolved through discussion with a third author (A.A).

## 3. Results

### 3.1. Study Selection

**Figure 1:**
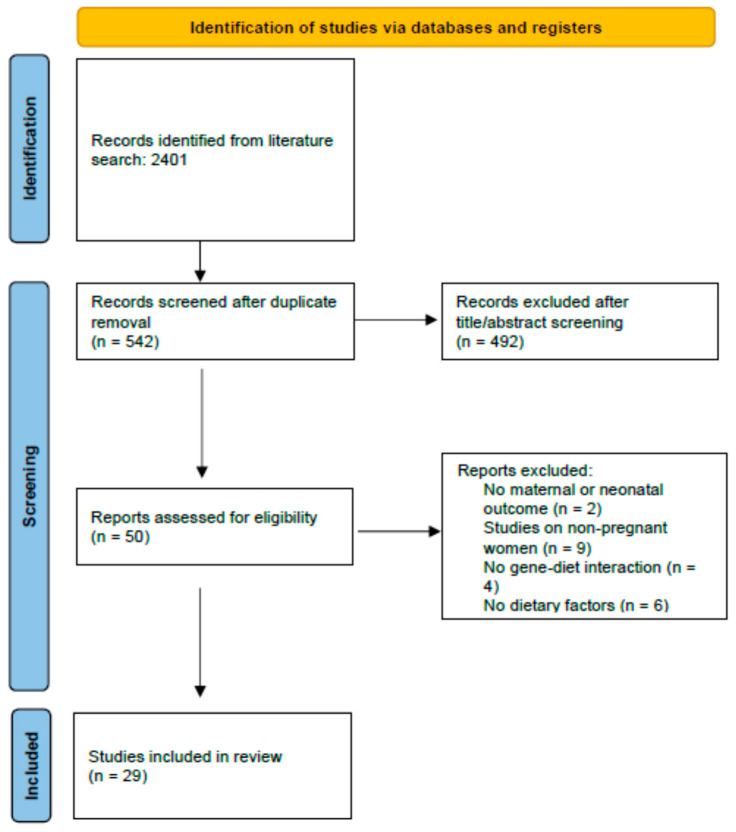
PRISMA flow diagram of study selection process.

Figure 1 illustrates the study selection process using the PRISMA flow diagram. After removing duplicate articles, a total of 542 unique articles were identified from the databases. Of these, 492 were excluded after screening their titles and/or abstracts. Following full-text screening, 21 studies were further excluded based on our selection criteria. These exclusions encompassed two studies that did not evaluate pregnancy outcomes, six studies that did not evaluate dietary factors, nine that did not consider a study population in line with our objectives, and four studies that did not evaluate the interplay between maternal dietary habits, genetic variants, and adverse pregnancy outcomes. Thus, a total of 29 articles are included in the present systematic review. Table 1 provides an overview of these studies, including details about their design, study population, the genetic variants explored, and their key findings.

### 3.2. Characteristics of Studies Investigating Maternal Outcomes

Eleven studies focused on investigating the interaction between genetic variations, maternal dietary factors, and pregnancy outcomes. These studies were all published between 2017 and 2023, with the majority conducted in China (*n* = 7). Additionally, four studies were conducted in various regions, including Russia, United States (USA), and Spain. Among these studies, six adopted a retrospective study design, while three were prospective. The remaining articles included one survey and one randomized controlled trial. All included studies were conducted on pregnant women and/or their infants, for whom SNP analysis was performed. In the following sections, we present the findings of these studies, organized according to the specific pregnancy outcome they investigated.

#### 3.2.1. Gestational Diabetes Mellitus

Women with GDM are exposed to higher risk of experiencing adverse perinatal and neonatal consequences, including gestational hypertension [53], PTB [54], and the development of cardiovascular diseases [55]. While some risk factors for GDM have been identified, the complete understanding of its etiology remains incomplete [56,57]. For these reasons, unveiling interactions between genetic, metabolic factors, as well as maternal exposures involved in the development of GDM is still challenging. Our review includes six studies exploring the interaction between genetic variants and dietary factors on the risk of GDM. Overall, these studies considered 49 SNPs.

Mo and colleagues explored the potential interaction between genetic variants in genes associated with vitamin D and glucose metabolism pathways, examining how these variants may interact with plasma 25(OH)D concentrations and influence the development of GDM. The authors found associations between 25(OH)D levels in the initial trimester of pregnancy and the CT genotype in CYP3A4, as well as the TT genotype in LRP2—both enzymes implicated in the regulation of vitamin D metabolism. These genetic variants were associated with an increased susceptibility to GDM [29]. Zhu and colleagues faced a comparable challenge. Their objective was to investigate the links between vitamin D concentrations in early pregnancy, genetic variations in vitamin D metabolic genes, and their impact on the susceptibility to GDM. While the authors identified a noteworthy association between two VDR gene variants, namely rs1544410 and rs731236, and susceptibility to GDM, they did not observe any interactions between genetic variants and vitamin D concentrations in relation to GDM risk [30].

In contrast, Popova and colleagues unearthed an interactive effect between maternal sausage consumption and the presence of risk alleles in two specific SNPs—rs10830963 in MTNR1B and rs1799884 in glucokinase gene (GCK)—pertaining to GDM risk. Intriguingly, the risk of GDM exhibited an upward trend with an increasing number of risk alleles, particularly among women with low to moderate sausage consumption [25]. Ao and colleagues highlighted a gene–diet interplay through a case–control study investigating the GCK association between the diet and pre-pregnancy sweets consumption in relation to GDM. The study revealed an interaction between the rs4607517 A allele of GCK and sweets consumption concerning GDM. Specifically, the presence of the GCK rs4607517 A allele increased the risk of GDM among women who regularly consumed sweets more than once a week [27]. In a different case–control investigation, Wang and colleagues sought to determine whether genetic variations in the CDKAL1 (cyclin-dependent kinase 5 regulatory subunit-associated protein1-like 1) gene, linked to proinsulin conversion and insulin resistance, in conjunction with low maternal serum levels of critical metabolic and nutritional factors (specifically, L-carnitine, choline, and betaine), could function as predictive markers for GDM [58]. The authors observed a noteworthy interaction associated with the risk of GDM between the CC and CG genotypes of the rs7747752 polymorphism and low serum levels of L-carnitine and/or choline [26].

Finally, Barabash and colleagues uncovered an interaction between the TCF7L2 gene polymorphism, responsible for regulating glucose homeostasis, and maternal adherence to a Mediterranean diet in relation to the risk of developing GDM. Women carrying the rs7903146 T-allele, who followed a Mediterranean diet in the early stages of pregnancy, demonstrated a reduced risk of GDM compared to those with the CC variant [28].

#### 3.2.2. Other Maternal Outcomes

Five studies explored possible interactions between maternal nutritional factors and SNPs in genes linked to metabolic pathways, investigating their association with HDP, RSA, RPL, IDA, and GWG. A total of 58 SNPs were considered across these studies.

The increasing prevalence of HDP is a significant factor contributing to almost 20% of worldwide maternal mortality, elevating the associated risks for maternal, child, and long-term cardiovascular health [59,60,61]. The existing research has predominantly focused on exploring the relationship between vitamin D status, gene variations in the vitamin D metabolic pathway, and HDP, frequently neglecting possible interactions among these factors [62,63]. For instance, Si and colleagues investigated the interplay between maternal SNPs in genes related to the vitamin D metabolic pathway (GC, CYP24A1, CYP3A4, CYP27B1, LRP2, and VDR) and vitamin D levels during pregnancy, examining their impact on gestational blood pressure and HDP development. Specifically, the study revealed that vitamin D exhibited interactions with polymorphisms in the CYP24A1, GC, and VDR genes, influencing blood pressure outcomes. Moreover, individuals with polymorphisms in CYP24A1 (rs2248137) and LRP2 (rs2389557 and rs4667591), coupled with vitamin D deficiency in the second trimester, demonstrated an elevated risk of developing HDP [33].

The occurrence of a minimum of two terminated pregnancies before the 28th week of gestation, defined as RSA, represents another form of adverse pregnancy outcome. The exploration of immune system function abnormalities has been proposed as a potential avenue for understanding cases of unexplained infertility associated with RSA [64]. In this context, Wang and colleagues investigated the interplay between environmental factors and the CD28/B7 pathway in RSA. The study revealed that individuals with the rs1915087 TT, rs6804441 GG, or rs41271391 TT genotypes, in combination with vitamin supplements, experienced a significantly reduced risk of RSA. Furthermore, women who regularly consumed fresh vegetables and fruits, especially those with the rs3116496 TT genotype, were less likely to develop RSA compared to those who had limited intake of fresh foods. Conversely, the rs3181098 AA and rs3181100 CC genotypes were strongly associated with an increased prevalence of RSA, particularly among individuals with low vegetable and fruit consumption [35].

Elevated homocysteine levels, often associated with the MTHFR C677T polymorphism, serve as a catalyst for vascular inflammation, prompting the formation of consequential microemboli at the maternal–fetal interface. Simultaneously, women with MTHFR 677TT also have diminished levels of vitamin D that contribute to heightened natural killer (NK) cell cytotoxicity, intensifying inflammatory immune responses at the maternal–fetal interface and thereby causing RPL. In a parallel investigative effort, Ota and collaborators delved into the intricate interplay between 25(OH) vitamin D, homocysteine levels, and the MTHFR C677T polymorphism in women experiencing RPL. The authors aimed to assess the impact of the MTHFR C677T polymorphism on homocysteine and vitamin D levels, along with immune parameters (i.e., NK cell cytotoxicity). Notably, their findings shed light on the nuanced relationship between nutritional factors and immune responses in the context of RPL, unveiling an inverse correlation between vitamin D levels and homocysteine, particularly among individuals carrying the TT genotype. Furthermore, the research showed the independent effect of vitamin D deficiency, suggesting its role in heightening susceptibility to hyperhomocysteinemia [34].

Iron deficiency during pregnancy, encompassing both gestational iron deficiency and IDA, represents a widespread nutritional challenge globally [65]. From a genetic point of view, some studies have indicated that the polymorphism of the haptoglobin (Hp) gene could potentially influence the interplay among dietary factors, nutrient metabolism, and the susceptibility to nutritional disorders [65,66]. With this purpose, Hu and collaborators sought to investigate whether SNPs of Hp—a scavenger of free hemoglobin (Hb) that impacts blood iron levels—could potentially alter the relationship between dietary iron intake and the risk of gestational IDA [31,67]. The research uncovered a significant interaction between genetic factors and dietary influences on serum ferritin levels among carriers of the Hp 1 and Hp 2 alleles. Pregnant women with the Hp 1-1 genotype who fell short of the recommended dietary allowance of iron or lacked a normal blood iron status, as well as those who did not utilize erythropoiesis-related total prenatal supplements, exhibited an elevated risk of developing gestational IDA [31].

Multiple studies have hinted at the potential influence of particular genes linked to obesity risk (i.e., FTO, MC4R, and TMEM18) on GWG, emphasizing the necessity for in-depth investigations into their roles [32,68]. Given this perspective, Meng and collaborators demonstrated how gene–diet interactions impact GWG. They emphasized that the influence of the KCTD15 gene on GWG could be modified by dietary fat intake. The authors identified a noteworthy interaction involving the rs11084753 polymorphism near the KCTD15 gene, dietary fat consumption, and GWG in individuals with the AG genotype. Women with this genotype experienced increased weight gain during pregnancy when they had a higher fat consumption [32].

### 3.3. Characteristics of Studies Investigating Neonatal Outcomes

In the period from 1998 to 2022, 18 studies investigated the relationships among SNPs, maternal dietary intake, and outcomes in neonates. Most of them were conducted in China (*n* = 6), followed by the USA (*n* = 2), South Korea (*n* = 2), Mexico (*n* = 2) and Indonesia (*n* = 2). Four studies were conducted in the Netherlands, Malaysia, New Zealand, and Sweden, respectively. Nine studies employed a retrospective study design, eight employed a prospective approach, and one followed a cross-sectional methodology. All included studies were conducted on pregnant women and/or their infants, for whom SNP analysis was performed. In the following sections, we present the findings of these studies, organized according to the specific neonatal outcome they investigated.

#### 3.3.1. Anthropometric Measures

Six studies explored the influence of maternal diet and genetic variations on diverse anthropometric measurements at birth. Notably, a substantial emphasis was placed on infants categorized as SGA, influenced by a combination of factors, including maternal socio-demographic characteristics, nutritional intake and status, and genetic factors [14,21,69,70]. Bulloch and colleagues explored the interplay between maternal one-carbon SNPs (MTHFR 677 (rs1801133), MTHFR 1298 (rs1801131), MTHFD1 1958 (rs2236225), MTR 2756 (rs1805087), MTRR 66 (rs1801394), and TCN2 776 (rs1801198)) and the use of folic acid supplements (FASs) in relation to the risk of SGA births. Their findings revealed a significant interaction between specific SNPs (MTHFR 1298, MTHFR 677, and TCN2 776) and FASs when it comes to SGA. Notably, women who did not use FASs and carried variant alleles of MTHFR 1298, MTHFR 677, or TCN2 776 had a higher likelihood of SGA. No significant interactions were observed for MTHFD1 1958, MTR 2756, or MTRR 66 [21].

Chun and colleagues demonstrated an interaction between maternal genetic variations in the GC gene, responsible for encoding the vitamin-D-binding protein, 25(OH)D concentrations in maternal and umbilical cord blood, and infant birth weight. They found that low 25(OH)D levels in both maternal and cord blood were significantly linked to reduced birth weight in infants born to mothers carrying the rs12512631 SNP. In addition, low 25(OH)D levels in cord blood were notably associated with decreased birth weight only in infants born to mothers carrying the rs7041 allele [37].

In a similar vein, Lee and colleagues investigated the impact of maternal and neonatal plasma 25(OH)D concentrations and the VDR (i.e., rs2228570) and GC SNPs (i.e., rs4588 and rs7041) on neonatal birth measurements. The authors found that neonates born to mothers with the VDR rs2228570 GG genotype and with vitamin D deficiency had significantly larger head circumferences [36].

The research led by Aji and collaborators delved into the interaction involving a genetic risk score (GRS) derived from six SNPs within VDR genes, maternal 25(OH)D levels, and neonatal anthropometric measures. They identified an interaction between the GRS and 25(OH)D levels, particularly in relation to head circumference. The findings showed that mothers of infants with head circumferences less than 35 cm exhibited significantly lower 25(OH)D levels if they carried four or more risk alleles, as opposed to those with three or fewer risk alleles [40].

Interestingly, within the same cohort of mothers and children, the authors discovered a noteworthy interaction. This interaction pertained to a two-SNP GRS, specifically involving rs2228570 and rs7975232, derived from SNPs within VDR genes (VDR-GRS), and its interplay with maternal carbohydrate intake. Notably, this interaction was shown to have a discernible impact on outcomes related to birth length. In fact, pregnant women with elevated carbohydrate intake and two or more risk alleles of VDR-GRS had newborns with considerably shorter birth lengths in comparison to infants born to mothers with fewer than two risk alleles [38].

Torres-Sanchez and colleagues noted that insufficient maternal dietary vitamin B12 intake was significantly associated with decreased infant length and length for age at birth, particularly in infants born to mothers with the MTHFR C677T SNP. However, no association was observed between maternal dietary intake of folate and neonatal anthropometric measures [39].

#### 3.3.2. Preterm Birth

Two studies investigated the relationship between maternal levels of vitamins and/or minerals during pregnancy and the risk of PTB. Additionally, they examined whether a total of 19 maternal SNPs could influence this association.

Wang and colleagues explored the link between vitamin D levels during pregnancy, SNPs related to the synthesis and metabolism of vitamin D, and their association with gestational weeks and the risk of PTB. Notably, women harboring SNPs of the GC gene (rs16846876 and rs7041) and encountering vitamin D deficiency (VDD) in the second trimester witnessed a reduction in gestational weeks. Furthermore, those carrying the SNP of the VDR gene (rs4334089) and experiencing VDD in the third trimester had shorter gestational periods. A similar pattern was observed when examining interactions between GC (rs7041) and vitamin D regarding the risk of spontaneous preterm birth (SPB) [41].

Hao and colleagues investigated the relationship between maternal Mn levels, the risk of PTB in early pregnancy, and SNPs in the genes encoding superoxide dismutase (SOD) and catalase (CAT). Their study revealed that women exposed to high Mn levels and possessing the AA and AG variants of rs2758352, the CC variant of rs699473, and the GG variant of rs769214 were more likely to experience a PTB [42].

#### 3.3.3. Abnormal Embryonic Development

Eight studies explored the impact of maternal SNPs on the relationship between maternal diet and conditions associated with abnormal embryonic development. Congenital heart disease (CHD), one of the most prevalent birth defects, involves structural or functional abnormalities resulting from the underdevelopment of the heart and major blood vessels during early embryonic development. These conditions are influenced by intricate interactions between genetic variations and environmental factors [71,72]. Hence, Li and collaborators postulated putative interactions between maternal dietary factors and genetic variants of cystathionine beta synthase (CBS)—a pivotal enzyme gene in the folate metabolic pathway. These interactions were thought to collectively contribute to the development of CHDs in offspring. In essence, if mothers possessed a risk genotype at rs2851391 or rs234714, then the probability of their offspring developing CHD significantly rose when exposed to a diet rich in unhealthy foods [45]. As accumulating evidence suggests that periconceptional folic acid supplementation could be preventive against CHD, Van Beynum and co-authors demonstrated that maternal SNPs in methionine synthase reductase (MTRR), coupled with elevated plasma methylmalonic acid concentrations—an indicator of vitamin B12 deficiency—may be indicative of an elevated risk of CHD in offspring [46].

Gatica-Dominguez explored the intricate interplay between maternal nutritional factors, genetic variations, and their impact on the cognitive development of offspring. To do that, the authors conducted an exploration into the potential association between maternal levels of folate and vitamin B12 during pregnancy and the neuropsychological development of children, also considering the potential role of the maternal MTHFR C677T genotype in influencing this relationship. The findings revealed a significant interaction between maternal plasma folate and MTHFR C677T genotypes. Indeed, Mental Development Index (MDI) was inversely associated with maternal plasma folate in offspring born to women with the MTHFR 677CC genotype. No significant interactions were found between MDI, maternal vitamin B12, and MTHFR C677T genotypes [43].

In this context, the maternal periconceptional use of vitamin supplements containing folic acid has been shown to significantly diminish the risk of neural tube defects (NTDs) in offspring. Recent studies have further indicated an association between the C677T genotype of the MTHFR gene and the risk of NTDs. To explore potential interactions influencing the risk of spina bifida, Shaw and collaborators conducted a study investigating the interplay between the infant’s MTHFR C677T genotype and maternal usage of folic acid supplements. Among infants whose mothers did not use supplements containing folic acid during the periconceptional period, the risk of spina bifida was notably higher for those with the TT genotype compared to infants with the CC genotype [47].

Moreover, it has been shown that maternal genetic factors in the metabolic pathway of vitamin D are associated with type 1 diabetes in the child. Miettinen and colleagues analyzed the genetic determinants of 25(OH)D concentration during pregnancy in mothers whose children later developed type 1 diabetes. The connections between serum 25(OH)D concentration and SNPs in the VDR (rs4516035) and GC (rs12512631) genes were more pronounced in mothers whose children developed type 1 diabetes [48]. Song and colleagues aimed at assessing the association between maternal methylenetetrahydrofolate dehydrogenase 1 (MTHFD1) gene polymorphisms, maternal dietary habits in early pregnancy, and their interactions with the risk of ventricular septal defects (VSD) in offspring. By comparing mothers of VSD cases and mothers of healthy infants, they observed significant interaction effects between maternal dietary habits and genetic SNPs of the maternal MTHFD1 gene at rs1950902, rs2236225, and rs2236225 on the risk of VSD [49].

In a similar vein, Luo and colleagues investigated the relationship between genetic variations in the maternal betaine–homocysteine methyltransferase (BHMT) gene, maternal dietary patterns, and how they interacted to influence the risk of VSD in the offspring. Their findings indicated that mothers with variant genotypes who also reported regular or low consumption of beans during pregnancy were at a notably elevated risk of having offspring with VSD when compared to those with the wild genotype (TT) who also reported regular bean intake [50].

Finally, Guo and collaborators investigated the interplay between two genetic variations (i.e., C641A and G15572) within transforming growth factor beta 3 (TGFb3) and maternal exposures during pregnancy in relation to the occurrence of cleft lip with/without cleft palate (CL/P). Interestingly, although they evaluated different models, which also included maternal multivitamin supplements, the model including maternal passive smoking and TGFb3 C641A showed the best ability to predict the risk of CL/P. These findings suggested a synergistic effect of TGFb3 C641A and maternal passive smoking, also providing potential strategy for identifying sub-groups of individuals at higher risk of CL/P [44].

#### 3.3.4. Other Neonatal Outcomes

Two studies explored the influence of prenatal maternal diet on offspring disease susceptibility, considering a total of 958 maternal and/or neonatal SNPs. Mazul and colleagues proposed that SNPs in genes related to folate and choline in both offspring and mothers might be associated with neuroblastoma. They also suggested that these genetic factors could potentially interact with the maternal intake of folate, choline, and folic acid to influence the risk of neuroblastoma. Of particular interest, they observed interactions between maternal choline dietary intake and specific SNPs, including MTHFD1L (rs10489810) and TYMS (rs9966612) in the offspring. When maternal choline consumption was below the 25th percentile, offspring carrying the G allele of rs1738575 (MTHFD1L) had an increased risk of neuroblastoma, whereas those with the A allele in SNP rs9966612 (TYMS) had a lower risk of developing neuroblastoma [51].

Moreover, Hong and colleagues genotyped neonatal cord blood for SNPs of genes involved in the development of respiratory tract infection (RTI). Interestingly, the authors noted interaction effects between CD14, TLR4, and GSDMB SNPs and prenatal antioxidant intake on RTI risk in infants at 12 months of age [52]. Indeed, a higher prenatal maternal intake of vitamins A and C, folate, fruits, and total fruits and vegetables reduced the risk of RTI in infants with the TT genotype of CD14. Moreover, a significant interaction of the TLR4 genotype was only found with vitamin C. In terms of GSDMB rs4794820, the risk of RTI was reduced by a higher prenatal intake of fruits and vegetables in infants with the GA + AA genotypes in GSDMB [52].

## 4. Discussion

Our systematic review summarizes the current evidence describing how maternal genetic variants and diet converge to influence maternal–child pregnancy outcomes. By synthesizing information from 29 studies, our objective was to provide a comprehensive understanding of the intricate interactions between genetic variations and diet in the context of maternal and neonatal outcomes. The studies incorporated were published between 1998 and 2023, and their results reflect the dynamic and ongoing development within this area of study.

Research into gene–environment interactions can be instrumental in identifying particular subgroups of women and children who face an elevated risk of adverse health outcomes at birth [13]. Studying the interaction between genetic variants and maternal diet during the periconceptional period is of paramount importance both for maternal health and fetal development. This period is critical as it coincides with the early development of the fetus and plays a pivotal role in its future health trajectory. Maternal diet plays a key role in providing the necessary nutrients for fetal development, but individual genetics influence how the body processes and utilizes these nutrients. In this context, genetic variation is another crucial aspect. Each individual has genetic differences that affect their ability to absorb, metabolize, and use nutrients. Thus, understanding how genetic variants can influence women’s response to diet is essential to ensuring a healthy pregnancy and offspring well-being, as well as to identifying personalized dietary recommendations.

With respect to maternal outcomes, we noted that the majority of the studies explored whether maternal genetic variants may modulate the risk of GDM [29], one of the most common metabolic disorders during pregnancy. It is caused by the interaction between genetic predisposition and metabolic factors and is linked to unfavorable perinatal and neonatal consequences [73].

Recent research offers evidence that maternal VDR gene polymorphisms likely play a pivotal role by influencing the biological activity of vitamin D in certain unfavorable pregnancy outcomes [62,63]. The VDR gene encodes the vitamin D3 receptor, which belongs to the nuclear hormone receptor superfamily and plays a pivotal role in mediating the biological effects of vitamin D [74]. Notably, SNPs within this gene have been extensively studied and are frequently associated with various metabolic characteristics, suggesting that an examination of the genetic background could yield new insights into the role of the vitamin D metabolic pathway in the development of GDM [75].

Certain studies have also identified a significant interplay between SNPs within other genes associated with vitamin D metabolism, 25(OH)D levels, and the risk of GDM [29,30]. Additional research delved into maternal SNPs within genes involved in glucose metabolism, maternal dietary choices, and their connection to GDM risk [25,26,27,58]. It is important to highlight that, among these studies, only the one conducted by Barabash and colleagues considered maternal dietary habits, specifically the adherence to the Mediterranean diet, underscoring the necessity for further investigations to comprehensively explore the impact of maternal dietary patterns on adverse pregnancy outcomes [28]. In addition, we noted that the diverse maternal genotypes respond to environmental factors during pregnancy, shaping the overall impact on the risk of other maternal pregnancy outcomes [31,32,33,34,35,68].

With respect to neonatal outcomes, the areas investigated by studies included in our systematic review were the following: anthropometric measures, preterm birth, abnormal embryonic development, and other neonatal outcomes. On the contrary, few studies have explored the impact of interactions between folate genes and nutrients on SGA risk. Interestingly, the study of Bulloch and colleagues noted a significant gene–nutrient interaction in relation to SGA between maternal one-carbon metabolism gene polymorphisms (i.e., MTHFR 677, MTHFR 1298, and TCN2 776) and maternal folic acid supplement (FAS) use [21]. Throughout pregnancy, folate plays a pivotal role in fetal growth and development, underscoring the importance of maintaining optimal maternal folate status and homocysteine levels, with some biological mechanisms that may clarify these findings [76]. Both folate intake and polymorphisms in the folate-mediated homocysteine metabolic pathway influence maternal plasma folate and homocysteine levels [77]. These polymorphisms have the potential to disrupt folate metabolism, one-carbon metabolism, DNA synthesis, and methylation [78,79,80], posing a risk to fetal growth and development. The presence of these polymorphisms may necessitate an increased supply of folic acid for one-carbon metabolism [76]. Indeed, folic acid supplementation is likely to alleviate the impact of these SNPs on pregnancy outcomes. By providing additional folic acid, supplementation enhances the availability of one-carbon groups for the conversion of homocysteine to methionine, contributing to the maintenance of normal homocysteine levels [81,82,83].

Although the majority of studies reported conflicting results [37,84,85,86], we observed some associations between maternal vitamin D deficiency and larger head circumference and decreased birth weight. Any controversies in these associations may be attributed to methodological differences, encompassing the timing of maternal blood sampling, the defined cut-off values for vitamin D deficiency, and the analytical methods employed for 25(OH)D assessment. In this context, the interactive effect of maternal vitamin D deficiency with additional factors—such as pre-pregnancy BMI, infant sex, and GDM—need to be better understood [36,37].

Several studies have also explored the association between maternal levels of vitamins and/or minerals during pregnancy and the risk of PTB [41,42]. For instance, SNPs present in genes encoding antioxidant enzymes altered the association between maternal Mn levels and SPB. Pregnant women with specific SNPs in SOD and CAT were notably susceptible to the negative consequences of elevated maternal Mn levels. Excessive Mn has the potential to trigger oxidative stress and mitochondrial dysfunction by increasing mitochondrial reactive oxygen species (ROS), diminishing enzyme activity, and depleting the cellular defense mechanisms against antioxidants [87,88,89]. This dysfunction in mitochondria, in turn, amplifies the generation and accumulation of ROS, influencing the activity of SOD2 and escalating oxidative stress [88]. Moreover, the existing research indicates a connection between oxidative stress and a disrupted antioxidant system with the occurrence of SPB [90]. The increased production of ROS and diminished levels of enzyme activity and antioxidants have been identified in preterm placentas [90] and infants [91]. Consequently, an excess of Mn during pregnancy might contribute to SPB by instigating oxidative stress. To the best of our knowledge, this study is the most comprehensive systematic review investigating the impact of gene–diet interactions on maternal and neonatal outcomes. Our findings suggest that the increased maternal intake of essential vitamins and minerals, including folate and vitamins A, C, and D, may reduce the risk of adverse pregnancy outcomes such as preterm birth and respiratory tract infections.

Despite the growing interest in studying gene–diet interactions during the periconceptional period, it is noteworthy that many of the excluded studies in our analysis considered the three components separately, without examining their interaction [92]. One of the strengths of our study was its inclusion of a significant number of epidemiological studies employing an observational prospective study design.

However, it is important to take into account some limitations when interpreting the studies included in our systematic review. In fact, comparison between studies should be interpreted with caution due to the high heterogeneity that may account for discrepancies in the obtained results.

Firstly, a considerable number of studies utilized varied methodologies to evaluate maternal dietary patterns and nutritional aspects, which may have compromised the precision of dietary intake assessment and the applicability of the findings. Furthermore, our study did not investigate how adherence to alternative restrictive diets (such as vegan, raw vegan, gluten-free, ketogenic) and genetic polymorphisms might impact pregnancy outcomes. Hence, further research could enhance the comprehension of underlying mechanisms, consequently facilitating the formulation of more efficient strategies for managing pregnancy in women adhering to restrictive dietary patterns.

Second, the heterogeneity of data reported in the included studies hindered the possibility of synthesizing the results through a meta-analytical approach. In fact, the variety of molecular methods used in the primary studies for genotyping, along with differences in study populations, definitions of exposure and outcomes, and differences in the study designs, have made it challenging to pool the data to conduct a quantitative synthesis of the results. Additionally, the scarcity of available data on certain specific genes or dietary patterns in relation to mother–child health may have further contributed to limiting the possibility of conducting a meta-analysis. Third, most of the studies had limited small sample sizes, encompassing both pregnant women and infants, raising the need for future analyses. Moreover, it is noteworthy that a significant number of the studies included in the analysis encompassed diverse study populations concerning ethnicity. This etherogeneity poses a challenge to exploring whether interactions between genotype and diet could be influenced by specific racial or ethnic demographics. The fourth limitation pertains to the health outcomes explored in our work. Notably, for maternal health outcomes, the most extensively investigated outcome was GDM, underscoring the necessity for further research to explore the role of gene–diet interactions in both physiological and pathological health conditions during pregnancy. Lastly, the observed interactions could be influenced by additional maternal lifestyles and exposures, which were not considered in these studies.

## 5. Conclusions

Despite the growing interest in studying gene–diet interactions during the periconceptional period, the existing evidence of their implications in reducing the risk of future diseases in children is still weak. Research on gene–diet interactions provides the opportunity to tailor dietary recommendations based on the genetic profile of pregnant women, optimizing maternal–child health and ensuring they receive the nutrient intake they need based on their genetic profile. Thus, deeply understanding this interaction could be useful in developing highly personalized approaches to maternal and child nutrition, as well as in exploring the potential implications in disease prevention and the promotion of the long-term well-being of both mothers and their offspring.

## Figures and Tables

**Table 1:** Characteristics of studies included in the current systematic review.

First Author	Year	Study Design	Population Ethnicity	Population	Dietary Factor	Genetic Variant	Total SNPs	Molecular Analysis for SNP Genotyping	Sample	Outcome
Popova [25]	2017	Case–control	European	278 women with GDM and 179 controls	Dietary information using ad hoc questionnaires	SNPs in MTNR1B (rs10830963 and rs1387153), GCK (rs1799884), KCNJ11 (rs5219), IGF2BP2 (rs4402960), TCF7L2 (rs7903146 and rs12255372), CDKAL1 (rs7754840), IRS1 (rs1801278) and FTO (rs9939609)	10	FlexiGene DNA Kit (Qiagen, Hilden, Germany)	Blood	GDM
Wang [26]	2022	Case–control	Asian	243 GDM women and their pregnant women control	Serum levels of L-carnitine, choline, and betaine	SNP (rs7747752)	1	Illumina Infinium^®^ Global Screening Array (Illumina, London, UK)	Blood	GDM
Ao [27]	2020	Case–control	Asian	562 GDM cases and 453 controls	Sweets consumption using ad hoc questionnaires	GCK (rs4607517) polymorphism	1	Sequenom’s MassARRAY platform (Agena, San Diego, CA, USA)	Blood	GDM
Barabash [28]	2020	Randomized controlled trial	Mixed	874 pregnant women	Adherence to Mediterranean diet (MedDiet) using an ad hoc questionnaire	TCF7L2 (rs7903146) polymorphism	1	7500 Fast Real-Time PCR System (Applied Biosystems, Foster City, CA, USA)	Blood	GDM
Mo [29]	2021	Prospective cohort	Asian	2156 pregnant women	Plasma 25(OH)D2 and 25(OH)D3 concentrations	Vitamin-D-related SNPs (*CYP24A1*: rs2209314, *CYP3A4*: rs2242480, *GC*: rs1155563, rs16846876, rs17467825, rs2282679, rs2298849, rs2298850, rs3755967, rs4588, rs7041, *LRP2*: rs10210408 and *VDR*: rs10783219); GDM-related SNPs (CDKAL1: rs7754840, rs7754840 IGF2BP2: rs1470579; MTNR1B: rs10830962; PRKCE: rs11682804)	19	Sequenom MassARRAY iPLEX Gold platform (Sequenom, San Diego, CA, USA)	Blood	GDM
Zhu [30]	2019	Case–control	Asian	654 pregnant women (274 GDM cases and 380 age-matched controls were included)	Serum 25(OH)D Level	SNPs (rs1544410 and rs731236) in the VDR; rs2282679 and rs7041 in the vitamin-D-binding protein (DBP), rs3829251 in 7-dehydrocholesterol reductase (DHCR7); rs6013897 in the cytochrome P450 family 24 subfamily A member 1 (CYP24A1); rs6599638 in chromosome 10 open reading frame 88 (C10orf88)	7	Improved multiple ligase detection reaction (iMLDR)	Blood	GDM
Hu [31]	2023	Survey	Asian	1430 pregnant women	Erythropoiesis-related diets [dietary intake (e.g., iron, vitamin A, and vitamin C) and prenatal supplements] and serum ferritin concentrations	Hp SNPs	3	Hp phenotyping was conducted using native polyacrylamide gel electrophoresis	Blood	IDA
Meng [32]	2018	Prospective cohort	African American	85 pregnant women	Dietary information using 24 h recall	SNPs of obesity-risk genes (rs5443; rs9939609; rs17782313; rs11084753; rs7498665; rs2568958; rs10938397)	7	Not available	Saliva	GWG
Si [33]	2022	Prospective cohort	Asian	3699 pregnant women	Plasma 25(OH)D levels	SNPs in the VitD metabolic pathway (CYP27B1: rs10877012, CYP3A4: rs2242480, rs4646437, LRP2: rs4667591, rs10210408, rs2228171, rs7600336, rs2544381, rs2544390, rs2389557, GC: rs16846876, rs12512631, rs17467825, rs2070741, rs2282679, rs3755967, rs2298850, rs4588, rs7041, rs222020, rs1155563, rs2298849, VDR: rs2228570, rs7975232, rs11568820, rs2238136, rs2853559, rs4334089, rs10783219, CYP24A1: rs6013897, rs2762934, rs2209314, rs6127118, rs2248137)	34	Sequenom MassARRAY iPLEX Gold platform (Sequenom, San Diego, CA, USA)	Blood	HDP
Ota [34]	2020	Retrospective cross-sectional study	African American	837 women with RPL	25 (OH) vitamin D and total plasma homocysteine	MTHFR SNP (C677T)	1	Polymerase chain reaction and reverse hybridization using the MTHFR 677CT RealFastTM Assay (ViennaLab Diagnostics GmbH, Vienna, Austria)	Blood	RPL
Wang [35]	2017	Retrospective study	Asian	1320 pregnant women	Diet with questionnaires	SNPs of CD28 (rs3116496; rs3769684; rs3181098; rs3181100; rs4673259; rs10932017), B7-2 (rs1129055 rs17281995; rs1915087; rs9282641) and B7-1 (rs6804441; rs41271391; rs16829984) involved in immune system	13	PCR-RFLP	Blood	RSA
Lee [36]	2022	Cross-sectional study	Asian	217 mother–neonate dyads	Plasma 25(OH)D concentration was measured in maternal and umbilical cord blood	VDR SNP (rs2228570) and GC SNPs (rs4588 and rs7041)	3	High-resolution melting (HRM) and restriction fragment length polymorphism	Blood	Anthropometric measures at birth
Chun [37]	2016	Prospective	Asian	356 pregnant women and their infants	25(OH)D levels in maternal and umbilical cord blood	GC SNPs (rs12512631, rs17467825, rs2282679, rs2298850, rs7041, rs1155563)	6	ABI PRISM SNaPShot multiplex kit (ABI, Foster City, CA, USA)	Blood	Birth weight
Aji [38]	2022	Prospective cohort	Asian	183 pregnant women and their newborns	25(OH)D levels in maternal blood and dietary information using FFQ	DHCR7 (rs12785878), CYP2R1 (rs12794714), GC (rs2282679), CYP24A1 (rs6013897) and VDR (rs2228570 and rs7975232)	6	Competitive allele-specific PCR-KASP assay	Blood	Anthropometric measures at birth
Torres-Sanchez [39]	2014	Prospective cohort	Hispanic	231 pregnant women	Dietary information using FFQ	MTHFR SNPs (C677T and A1298C)	2	PCR-RFLP	Blood	Pregnancy maternal outcomes and anthropometric measures at birth
Aji [40]	2020	Prospective cohort	Asian	183 pregnant women	25(OH)D serum level	DHCR7 (rs12785878), CYP2R1 (rs12794714), GC (rs2282679), CYP24A1 (rs6013897), and VDR (rs2228570 and rs7975232)	6	Genotyping was performed at LGC Genomics, London, UK	Blood	Pregnancy maternal outcomes and anthropometric measures at birth
Bulloch [21]	2020	Prospective cohort	European	2002 pregnant women	FAS use with questionnaire	MTHFR 677 (rs1801133), MTHFR 1298 (rs1801131), MTHFD1 1958 (rs2236225), MTR 2756 (rs1805087), MTRR 66 (rs1801394), TCN2 776 (rs1801198)	6	Multiplex genotyping using the Sequenom Mass Array System	Blood	SGA
Wang [41]	2021	Prospective cohort	Asian	3465 pregnant women	25(OH)D concentration	GC (rs16846876, rs17467825, rs2282679, rs3755967, rs2298850, rs4588, rs7041, rs1155563, rs2298849), CYP24A1 (rs2209314, rs6127118, rs2248137), CYP27B1 (rs10877012), LRP2 (rs10210408, rs2228171), and VDR (rs10783219)	16	Sequenom MassARRAY iPLEX Gold platform (Sequenom, San Diego, CA, USA).	Blood	PTB and gestational week
Hao [42]	2020	Nested case–control study	Asian	528 pregnant women (147 cases of SPB and 381 controls)	Maternal serum concentration of manganese level	SOD2 (rs2758352), SOD3 (rs699473), CAT (rs769214)	3	Not available	Blood	SPB
Gatica-Dominguez [43]	2020	Prospective cohort	Hispanic	181 mother–child dyads	Maternal plasma folate and vitamin B12	MTHFR SNP (C677T)	1	PCR	Blood	Child neuropsychological development
Guo [44]	2010	Case–control	Asian	Not available	Maternal multivitamin use	TGFβ3 neonatal SNPs (C641A and G15572)	2	Not available	Not available	CL/P
Li [45]	2020	Case–control	Asian	464 mothers with CHD infants and 504 control mothers	Maternal dietary intake	CBS SNPs (rs12613, rs234783, rs234784, rs2851391, rs2298759, rs234785, rs234713, rs234714 and rs1051319)	9	Not available	Blood	CHD
van Beynum [46]	2011	Case–control	European	169 CHD patients and 213 child controls, and 159 mothers with a CHD-affected child and 245 female controls	Plasma methylmalonic acid concentrations	MTRR SNP (MTRR 66A > G)	1	Not available	Blood	CHD
Shaw [47]	1998	Case–control	Mixed	214 liveborn case infants with spina bifida and 503 control infants	Maternal periconceptional use of supplements containing folic acid with questionnaire	Infant MTHFR SNP (C677T)	1	PCR	Newborn blood	Spina bifida
Miettinen [48]	2017	Case–control	European	474 mothers of type 1 diabetic children and 348 mothers of non-diabetic children	Serum 25(OH)D concentration during pregnancy	SNPs in NADSYN1/DHCR7 (rs4945008), VDR (rs154410, rs4516035, rs10783219), GC (rs12512631, rs4588), and CYP27A1 (rs17470271) genes	7	TaqMan (Applied Biosystems, Paisley, UK)	Saliva	Type 1 diabetes
Song [49]	2022	Case–control	Asian	360 mothers of VSD cases and 504 mothers of healthy infants	Dietary information using questionnaire	MTHFD1 SNPs (rs1950902, rs2236225, and rs2236222)	3	MassARRAY system (Agena iPLEX assay, San Diego, CA, USA).	Blood	VSD
Luo [50]	2022	Case–control	Asian	426 mothers of VSD children and 740 control mothers	Maternal dietary habits using FFQ	BHMT SNPs (rs3733890, rs1316753, rs567754, and rs1915706)	4	MassARRAY system (Agena iPLEX assay, San Diego, CA, USA	Blood	VSD
Mazul [51]	2016	Case–control	American	563 affected children and their parents	Pre-pregnancy supplementation and usual maternal dietary intake of folate, choline and folic acid with questionnaires	693 SNPs in 38 folate-related and 302 SNPs in 19 choline-related genes	958	GoldenGate Assay with the Illumina BeadStation 500GX Genetic Analysis System (Illumina, London, UK).	Saliva	Neuroblastoma
Hong [52]	2017	Prospective cohort	Asian	550 infants at 12 ages	Prenatal maternal diet with FFQ	CD14 (rs2569190), TLR4 (rs1927911), and GSDMB (rs4794820)	3	TaqMan method	Infant cord blood	RTI

## Data Availability

Data are contained within the article and Supplementary Materials.

## Supplementary Materials

The following supporting information can be downloaded at: https://www.mdpi.com/article/10.3390/nu16070994/s1, Table S1: PRISMA checklist.
